# Integrative analysis of rumen microbiota activity and host metabolism following methanogenesis inhibition in dairy cattle

**DOI:** 10.1128/spectrum.00269-26

**Published:** 2026-07-23

**Authors:** Simon Roques, Jérémy Tournayre, Paul S. Dou, Bénedict Yanibada, Hamid Boudra, Milka Popova, Diego P. Morgavi

**Affiliations:** 1 Université Clermont Auvergne, INRAE, VetAgro Sup, UMR Herbivores 481410, Saint-Genes-Champanelle, France; Foshan University, Foshan, China

**Keywords:** methanogenesis inhibition, methane, host-microbiota, multi-omics

## Abstract

Enteric methane emission from dairy cattle is an environmental challenge. The most efficient mitigation strategies nowadays include the use of methanogenesis inhibitors that specifically target the rumen methanogens. Specific inhibitors, such as 3-nitrooxypropanol (3-NOP), reduce methane emissions without negative effects on the products of fermentation that serve as energy metabolites for the host. However, the concomitant effects of methanogenesis inhibition on rumen microbiota and host metabolism are poorly characterized. Thus, the objective of this study was to explore the association between rumen microbiota and host metabolism when methanogenesis is inhibited. Thirteen dairy cows were used as controls, and 12 were supplemented with 3-NOP for 6 weeks. Rumen microbiota composition and activity were characterized using metagenomics and metatranscriptomics. The host metabolism was assessed in a previous publication by a metabolomic analysis of the plasma. Microbiota data were used as explanatory variables of the metabolome data in a multiblock sparse partial least squares analysis. Overall, the association between rumen microbiota and host metabolism was moderate. Notwithstanding this, a few downregulated transcripts related to glycolysis, hydrogen transfer, and protein synthesis, together with a decrease in the proportion of taxa of the Oscillospirales order, showed a correlation with host one-carbon metabolites (|r| > 0.6). These associations raised novel hypotheses that remain to be elucidated, especially with regard to the effects of dihydrogen on the accumulation of microbial glycolysis and methanogenesis metabolite intermediates.

**IMPORTANCE:**

Dairy cattle produce a substantial amount of methane, a potent greenhouse gas. Several strategies have been designed to reduce methane production by targeting the rumen microbiota. One such strategy specifically inhibits methanogens with a molecule called 3-nitrooxypropanol. This study uses an integrative data analysis approach, combining rumen microbiota and host metabolome information, to explore the consequences of inhibiting methanogenesis on the holobiont. This provides additional holistic insight into the effect of methane mitigation strategies on dairy cattle.

## INTRODUCTION

Methane emissions from livestock are an environmental concern that also hinders public acceptance of livestock production. Indeed, methane is a potent greenhouse gas with a warming effect of 86 times greater than that of carbon dioxide (CO_2_) over a 20-year period (1). The amount of methane emitted to the atmosphere has been increasing continuously worldwide since 1990. The livestock sector accounts for 45% of these emissions (2, 3), which puts the sector under great pressure to reduce the enteric methane emissions from the ruminants. In addition, enteric methane represents a loss of 6.3% ± 2% of the feed energy not converted into milk or meat (4). Although methane mitigation is often expected to improve feed efficiency, consistent improvements in animal productivity have not always been observed (4, 5). This discrepancy cannot currently be explained, in part, because the relationship between rumen microbiota and host metabolism has not been comprehensively explored when methanogenesis is inhibited. Specific inhibitors, such as 3-NOP, target methanogens (6), without modifying the nutritional value of cattle diets, which allows the effects of methanogenesis inhibition to be studied without the confounding effects of nutrition.

Methane in the rumen is produced by methanogenic archaea. These archaea can synthesize methane by three pathways, namely the hydrogenotrophic, the methylohydrogenotrophic, and the aceticlastic pathways (7, 8), the first two being the main ones in the rumen and using dihydrogen (H_2_) as an electron donor to reduce CO_2_ or methyl groups, respectively. All these pathways converge on the final MCR-catalyzed step, in which methyl-coenzyme M is reduced to methane. The 3-NOP inhibits the activity of MCR, an enzyme exclusively synthesized by methanogenic archaea (6, 8). Therefore, the specificity of 3-NOP for MCR does not directly affect the other microorganisms in the rumen, including bacteria that produce volatile fatty acids (VFA). Maintaining the production of VFA is a major prerequisite of any methane mitigation strategy, as the VFA represents 70% of the energetic supply to the host (9). These VFA are synthesized in the rumen by the breakdown of hexose to pyruvate, which is further fermented into the main VFA, acetate, butyrate, and propionate. The production of acetate and butyrate results in a net H_2_ production, whereas the production of propionate results in a net consumption of H_2_ (10). Given that 3-NOP inhibits the final reduction step in the methanogenesis pathway, the H_2_ not used in the production of methane may accumulate and would prevent the re-oxidation of NADH to NAD^+^ (5, 11), the latter being used in microbial glycolysis.

The effects of methane mitigation with 3-NOP on the production traits in dairy cows have been well documented (12), although the mechanistic links between methane reduction, microbial metabolism, and host metabolism that lead to changes in these production traits are scarcely described. At the host level, methane mitigation with 3-NOP slightly decreases dry matter intake and milk yield but increases milk fat, true protein, and milk urea nitrogen (12). At the rumen level, the downstream effect of methane mitigation is a shift in rumen fermentation toward the use of alternative hydrogen sinks, particularly propionate (5). It is suggested that this shift in the VFA profile leads to the aforementioned differences in host production traits, notably due to the gluconeogenic potential of propionate (13), which could enhance nitrogen utilization for milk protein synthesis (14, 15). Changes in butyrate production may also influence rumen NH₃ absorption and nitrogen metabolism (16).

In a publication of our group, which the present study will complete, the inhibition of methanogenesis by 3-NOP led to higher amino acid content in the plasma (17). However, the association between the inhibition of methanogenesis, the rumen microbiota, and the host metabolism remains unclear. The objective of this study was to characterize the relationship between rumen microbiota and host metabolism when methanogenesis is inhibited with 3-NOP. The study aimed to assess whether subtle changes in rumen microbiota activity can be associated with host metabolism. Metagenomics and metatranscriptomics were characterized while metabolomic data (17) were reused to assess the relationship between rumen microbiota. The present study extends the analysis of metabolomic host data previously reported by integrating it with rumen microbial transcripts and taxa. This integrative approach aimed to interpret changes in microbial composition and function in relation to host metabolic responses and to provide new insight into the metabolic effects associated with methanogenesis inhibition.

## MATERIALS AND METHODS

### Animals, diets, and experimental design

The experiment was described in detail in Yanibada et al. (17, 18). Briefly, 25 lactating Holstein-Friesian primiparous cows, fed a total mixed ration of corn silage (30%), grass hay (30%), and concentrate (40%), were divided into two groups. The experimental group (3-NOP, *n* = 12) was administered 3-nitrooxypropanol (60 mg/kg DM of total mixed ration), while a control group (CONTROL, *n* = 13) was administered a placebo. The experiment lasted 6 weeks, including 4 weeks for adaptation and 2 weeks for sampling and measurements.

### Sampling and methane emission measurements

In week 5, before the morning meal, rumen fluid samples were collected from each cow. Rumen fluid samples (about 500 mL) were taken using esophageal stomach tubing. Two representative aliquots (~ 10 mL) were promptly snap-frozen in liquid nitrogen and kept at −80°C until microbial community analysis. Another fraction (0.8 mL) was combined with 0.5 mL of deproteinizing solution (4 mg/mL crotonic acid and 20 mg/mL metaphosphoric acid in 0.5 M HCl), maintained at 4°C for at least 2 h, and centrifuged (16,500 × *g* for 10 min at 4°C), and the supernatant was stored at ‒20°C before VFA analysis by gas chromatography (GC) (19) using Perkin Elmer Clarus 580, FID equipment (PerkinElmer, Inc., MA, USA). Blood samples were collected through the jugular vein using heparinized vacutainers (Greiner Bio-One, Kremsmünster, Austria) (10 mL), in the morning after milking and before feeding. Blood samples were then centrifuged (3,000 × *g*; 15 min; 4°C), and plasma was transferred into 1.5 mL polypropylene tubes and stored at −80°C until analysis.

In week 6, 16 randomly selected cows (eight per group) were moved from free stalls into respiration chambers for 4 days to quantify methane emissions, as described (20).

### Rumen microbiota characterization

#### DNA extraction and sequencing

Total genomic DNA was extracted from rumen contents using a Qiagen DNeasy PowerSoil Pro Kit (Courtaboeuf, France) according to the instructions provided by the manufacturer. The concentration and quality of the DNA were determined spectrophotometrically by measuring the A260/A280 on a Nanodrop spectrophotometer (Nanodrop Lite, France). The genomic DNA was diluted to a concentration of 40 ng/μL and sent to Edinburgh Genetics for Whole Genome Sequencing using an Illumina chemistry. Individual metagenomes were sequenced using an Illumina NovaSeq for PE150 with a target sequencing depth of 30 M PE reads (Table S1).

#### RNA extraction and sequencing

Frozen rumen fluid was homogenized to a fine frozen powder using a blender mill under liquid nitrogen. The RNA was extracted from roughly 60 mg of homogenized frozen powder by a Qiagen RNeasy Plus Kit (Qiagen, Courtaboeuf, France). The yield and purity of the RNA extract were determined using a Nanodrop 1000 spectrophotometer (Nanodrop Lite, France). The RNA integrity was controlled using an Agilent 2100 Bioanalyzer (Agilent Technologies, Santa Clara, CA). In order to eliminate any residual DNA traces, a total RNA sample (6 µg) was subjected to a DNA digestion process using a TURBO DNA-free Kit (Thermo Fisher Scientific, MA, USA). The lack of genomic DNA was confirmed by the absence of amplification in a qPCR assay using universal primers targeting the bacterial 16S rRNA gene. In a final purification step, the Zymo RNA Clean and Concentrate Kit (Zymo Research Corp, Irvine, CA, USA) was used. About 2 µg of isolated total RNA was sequenced using Illumina HiSeq 4000 PE100 technology at the McGill and Genome Quebec Innovation Centre. Sequencing of RNA produced an average of 86 ± 9 M reads (Table S2).

#### Post-processing of DNA and RNA reads

The DNA reads were processed using HUMAnN (v3.8) (21) and MetaPhlAn (v4.0.6). Reads of DNA of low quality (<Q30) and reads that would match the Homo sapiens (hg37dec_v0.1) and Bos taurus (ARS-UCD1.2) genomes were removed with KneadData (v0.12.0) (22). After this quality and contamination removal, 82.7% ± 1.9% of DNA reads were retained. Remaining reads were then processed with MetaPhlAn to obtain taxonomic identification with default parameters. Gene identification was performed with HUMAnN. Briefly, in the workflow, the genomes of identified taxa are used to build a pangenome of known species on which the reads align. Unaligned reads on this pangenome were aligned on the UniRef90 protein database. RNA reads were also processed with HUMAnN, but not MetaPhlan, as the purpose of metatranscriptomics was to identify function rather than taxonomy. Beforehand, the RNA reads of low quality and reads from Homo sapiens and Bos taurus were removed. Then, rRNA reads were also filtered with KneadData to only conserve mRNA reads from rumen microbiota. After removal of these reads, 88.34% ± 8.7% of RNA reads were conserved, of which 5.00% ± 0.6% were mRNA reads. The mRNA reads were further aligned to the previously defined pangenome and the UniRef90 protein database.

### Host metabolome characterization

The characterization of the host metabolome has been described in Yanibada et al. (17). Briefly, the metabolome was characterized by a combination of mass spectrometry (MS) and nuclear magnetic resonance (NMR). Discriminant signals found by orthogonal signal correction partial least square discriminant analysis (OSC-PLS-DA) were subsequently identified as metabolites with an in-house database or public databases, namely METLIN (23), Livestock Metabolome Database (24), and BMRB (25).

### Omics-data integration and statistics

#### Phenotypic measures

Methane emissions (g/d), methane intensity (g/kg milk), methane yield (g/kg dry matter intake [DMI]), milk yield (kg/d), and milk yield-to-DMI ratio were measured and tested as detailed in Yanibada et al. (17). The effect of 3-NOP on each volatile fatty acid and on the ratio of acetate to propionate was tested by a Student’s *t*-test.

#### Integration

Three data sets were used for data integration, namely microbial taxonomic data, metagenome-normalized metatranscriptomic data, and host metabolomics data. The taxonomic count table was first filtered for taxa that appeared at least once in less than 50% of the samples with the *phyloseq* package (26). The zeros that remained in the table were imputed by geometric Bayesian-multiplicative replacement to pseudo-count with the *zCompositions* package (27). These resulting counts were further transformed to centered-log-ratio with the *composition* package (28).

Metatranscriptomic data suffer from an inherent limit, as any change in microbial RNA transcripts cannot be dissociated from a change in microbial, and thus DNA, abundance (29). To mitigate this limitation, we adjusted the transcriptomic data by accounting for the corresponding gene abundances derived from metagenomic data using the *MTXmodel* package (29). First, both metatranscriptomic and metagenomic data were filtered to keep reads with a minimum abundance of one in at least 50% of the samples. Metatranscriptomic and metagenomic data were then transformed to centered-log-ratio. Transcript abundances were then modeled as a function of the corresponding gene abundances, and the residuals from these models were extracted and used as surrogate variables for subsequent analyses (29). This step builds a linear model of each transcript with the corresponding gene as the unique continuous covariate. Residuals are then calculated as the difference between predicted and observed values of transcripts. In this way, we remove the contribution of gene abundance to the variance of transcript abundance. Biologically, the modeled variables can be interpreted as a centered expression value of the transcript. The effect of 3-NOP on individual transcripts was tested within the *MTXmodel* package with the treatment factor added to the covariate gene to model each transcript.

A multiblock sparse partial least squares (MB-sPLS) regression was used to integrate the three omics data sets, processed as described above. One sample identified as an outlier during preliminary sPLS analysis was removed from all datasets prior to integration to avoid disproportionate influence on the covariance structure between datasets. *MixOmics* package (30) was used to compute the MB-sPLS analysis, with variables of each block centered and scaled to unit variance. The metabolomic data set (20 discriminant metabolites) was specified as the response block (Y), while the taxonomic composition and DNA-corrected metatranscriptomic data were used as explanatory blocks (X).

A 5-fold cross-validation procedure was applied to determine the optimal number of variables to select per block, as well as the appropriate number of components for prediction, based on the coefficient of determination (R²) of prediction, computed using the *metrica* package (31). Variables selected on the first component—identified as the most explanatory and sufficient to capture the underlying group structure, as supported by the highest R² obtained during cross-validation—were then merged into a correlation network. Only associations with an absolute Pearson correlation coefficient ≥0.6 (|r| ≥ 0.6) were retained. The resulting network was visualized using *Cytoscape* v3.10.3 (32). Centrality was expressed as a degree, that is, the number of edges per node.

Normalized transcripts that were retained after omics integration were contextualized into their respective microbial metabolic pathways identified based on the literature (33–35). The relative abundances in counts per million of normalized transcripts were clustered into enzyme-class (EC) number, and the effect of 3-NOP on each EC of the respective pathway was tested with a Student’s *t*-test.

## RESULTS

### Methane emissions decreased without affecting rumen fermentation

Methane emissions and milk production are presented in Table S3. As reported in our previous publication, daily methane emissions, methane intensity, and methane yield decreased by ~23% in treated cows (*P* < 0.05), with no changes in milk production. Volatile fatty acid concentration in the rumen is presented in Table 1. The total concentration of VFA was not affected by the supplementation of 3-NOP compared to the CONTROL cows. The concentration of propionate tended to be higher in cows supplemented with 3-NOP (*P* = 0.089), and the acetate to propionate ratio was significantly reduced (*P* = 0.001) compared to the CONTROL cows.

**TABLE 1:** Volatile fatty acids concentration (mM) in the rumen of lactating dairy cows fed without (CONTROL, *n* = 13) or with 3-NOP (3-NOP, *n* = 12)

	CONTROL			3-NOP			*P*-value
	Average		sd^*a*^	Average		sd	
Total VFA	128.51		29.83	137.04		30.22	0.513
Acetate	89.30		22.70	90.5		20.4	0.888
Propionate	21.30		7.08	26.1		6.3	0.089
Butyrate	12.30		3.25	13.8		3.55	0.300
Isobutyrate	2.11		0.47	2.31		0.68	0.410
Isovalerate	2.18		0.60	2.7		1.02	0.139
Valerate	0.98		0.29	1.27		0.50	0.095
Caproate	0.34		0.10	0.36		0.23	0.763
Acetate/propionate	4.17		0.03	3.44		0.04	0.001

^*a*^ sd: standard deviation.

### Ruminal microbial composition was comparable between the two groups

**Fig 1:**
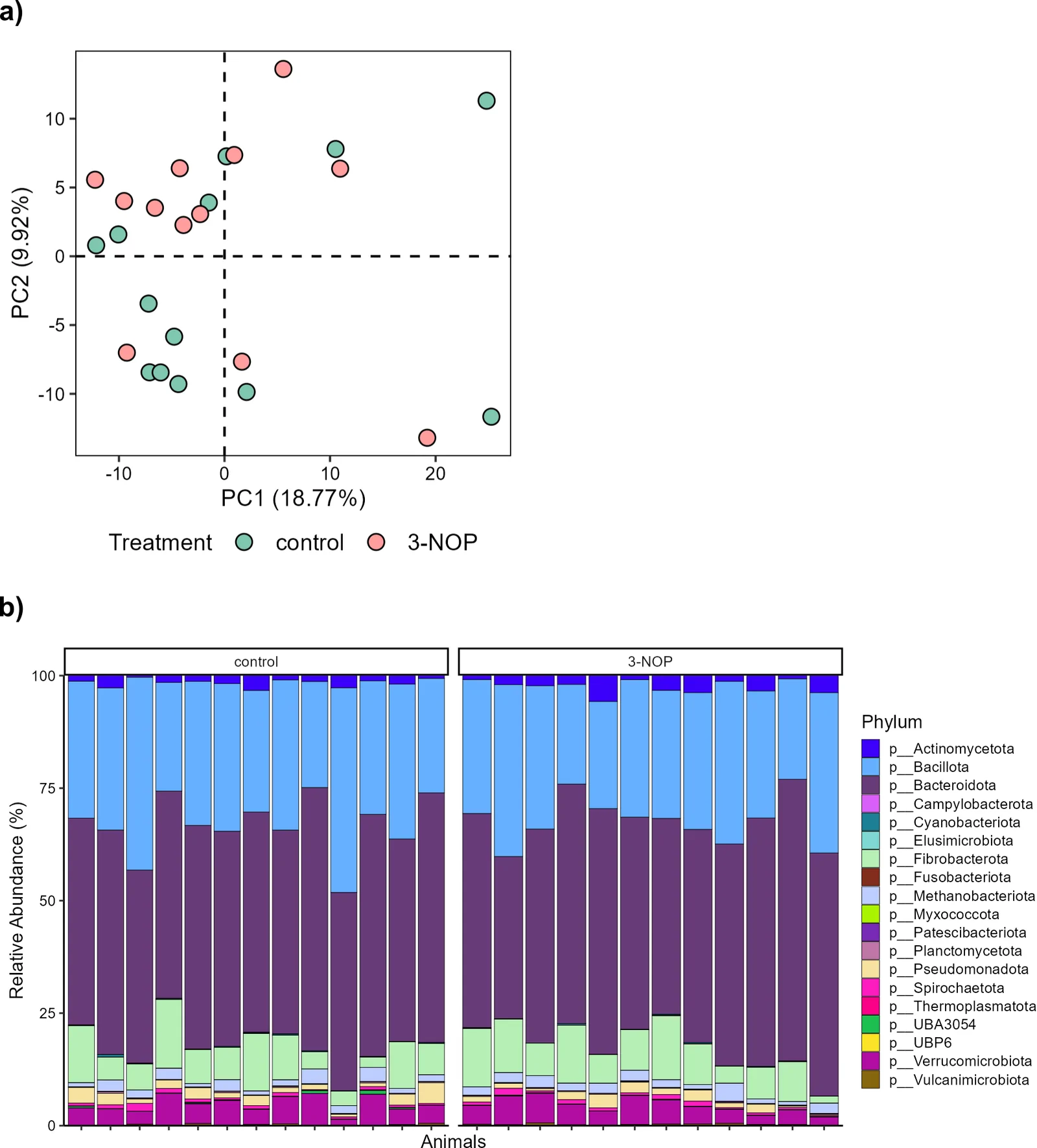
(**a**) Rumen bacterial community structure of dairy cows supplemented with 3-NOP (3-NOP; *n* = 12) or not (CONTROL, *n* = 13). Principal component analysis score plot of 607 taxa at the species level obtained after filtration for low abundance and clr-transformation. (**b**) Relative abundance of the rumen microbiota taxa at the phylum level grouped by treatment in the 25 dairy cows supplemented with 3-NOP (3-NOP) or not (CONTROL).

The rumen bacterial and archaeal community structure was similar between 3-NOP and CONTROL cows (Fig. 1a). The composition of the rumen bacterial community at the phylum level is presented in Fig. 1b. The five main phyla across the two groups were Bacteroidota (49.3% ± 5.67%), Bacillota (30.8% ± 5.93%), Fibrobacterota (8.31% ± 3.89%), Verrucomicrobiota (4.44% ± 1.7%), and Methanobacteriota (1.93% ± 0.85%). The proportion of archaea-affiliated sequences in the rumen microbiota of 3-NOP-supplemented cows (2.05% ± 0.9%) was similar to that of CONTROL cows (1.91% ± 0.9%, *P* = 0.64). In addition, the structure of the archaeal community was not different between CONTROL and 3-NOP cows, although only nine taxa remained after removing low-abundant taxa in the data set (Fig. S1). Eukaryota were not identified in the data set. Multi-omics data integration highlighted the relationship between microbial glycolysis and methanogenesis with host one-carbon metabolites.

Removing transcripts for low prevalence (less than 50% of samples) led to 13,260 remaining transcripts that were normalized by their corresponding genes, except for 810 transcripts that did not have a corresponding gene and were not normalized, resulting in 12,450 normalized transcripts used in the analysis. This exclusion may have removed some biological information and slightly narrowed the scope of the downstream analysis. However, these 810 transcripts represent only 6% of the retained transcripts, and subsequent analyses were therefore restricted to transcripts with matched gene-level information. The PCA of normalized transcripts showed no clear separation between the treatment groups (Fig. S2). A total of 17 transcripts were differentially expressed between 3-NOP-supplemented and control cows (FDR-adjusted *P* < 0.05; Table S4). These included several key methanogenesis-related enzymes—methyl-coenzyme M reductase subunits (beta and gamma), coenzyme B sulfoethylthiotransferase, and ferredoxin—all of which were less abundant in the 3-NOP group. Other downregulated features in the 3-NOP group were associated with translation (elongation factor Tu, ribosomal proteins), energy metabolism (glyceraldehyde-3-phosphate dehydrogenase), membrane phospholipid biosynthesis (CDP-diacylglycerol-glycerol-3-phosphate 3-phosphatidyltransferase), and chromatin structure (histone H4). Four UniProt entries were listed as redundant proteins in the whole UniProt database and were consequently not identified.

**Fig 2:**
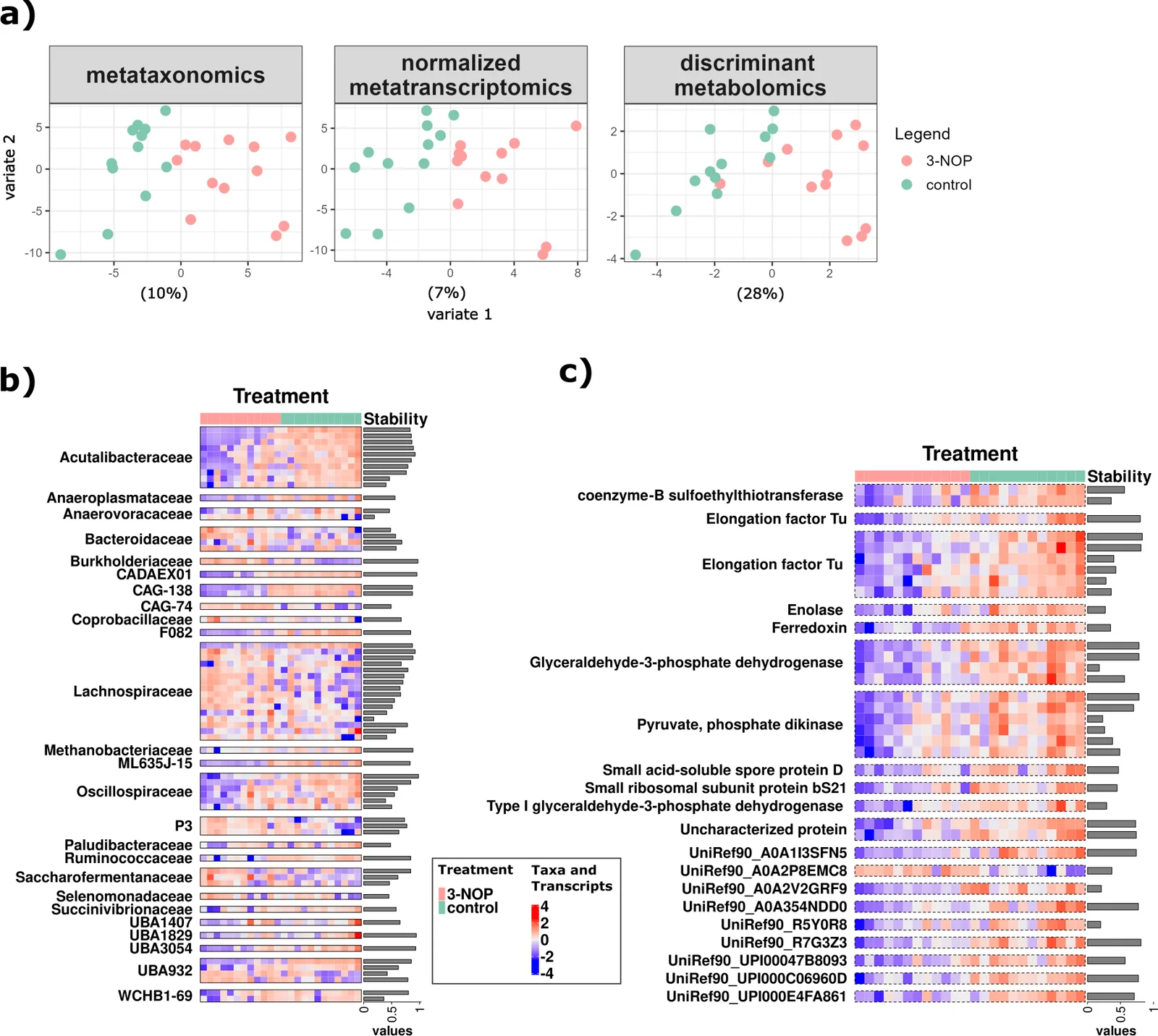
Multiblock sparse partial least squares analysis. (**a**) Scores plot of individuals on the first two components of the model for the explanatory blocks metataxonomics and normalized metatranscriptomics, and the target block discriminant metabolomics. The scores plot provides a low-dimensional representation of the integrated multi-omics structure and enables visualization of sample similarities and discrimination between experimental groups based on covariance patterns shared across omics layers. (**b**) Heatmap of selected features on the explanatory blocks metataxonomic and (**c**) normalized metatranscriptomic, on the first component. Features are listed in rows; they are unique, although multiple features are grouped by the same taxonomic name or the same transcript name. Animals are organized in columns and ordered by their first SPLS component scores. Red to blue color describes the scaled abundance of each feature in the MB-sPLS model. Unnamed features are deleted accession numbers from the UniProtKB database. Barplot on each graph describes the stability of the feature during cross-validation, that is, if the feature was retained in multiple rounds of cross-validation: 0, the features were not retained, and 1, the features were always retained.

**Fig 3:**
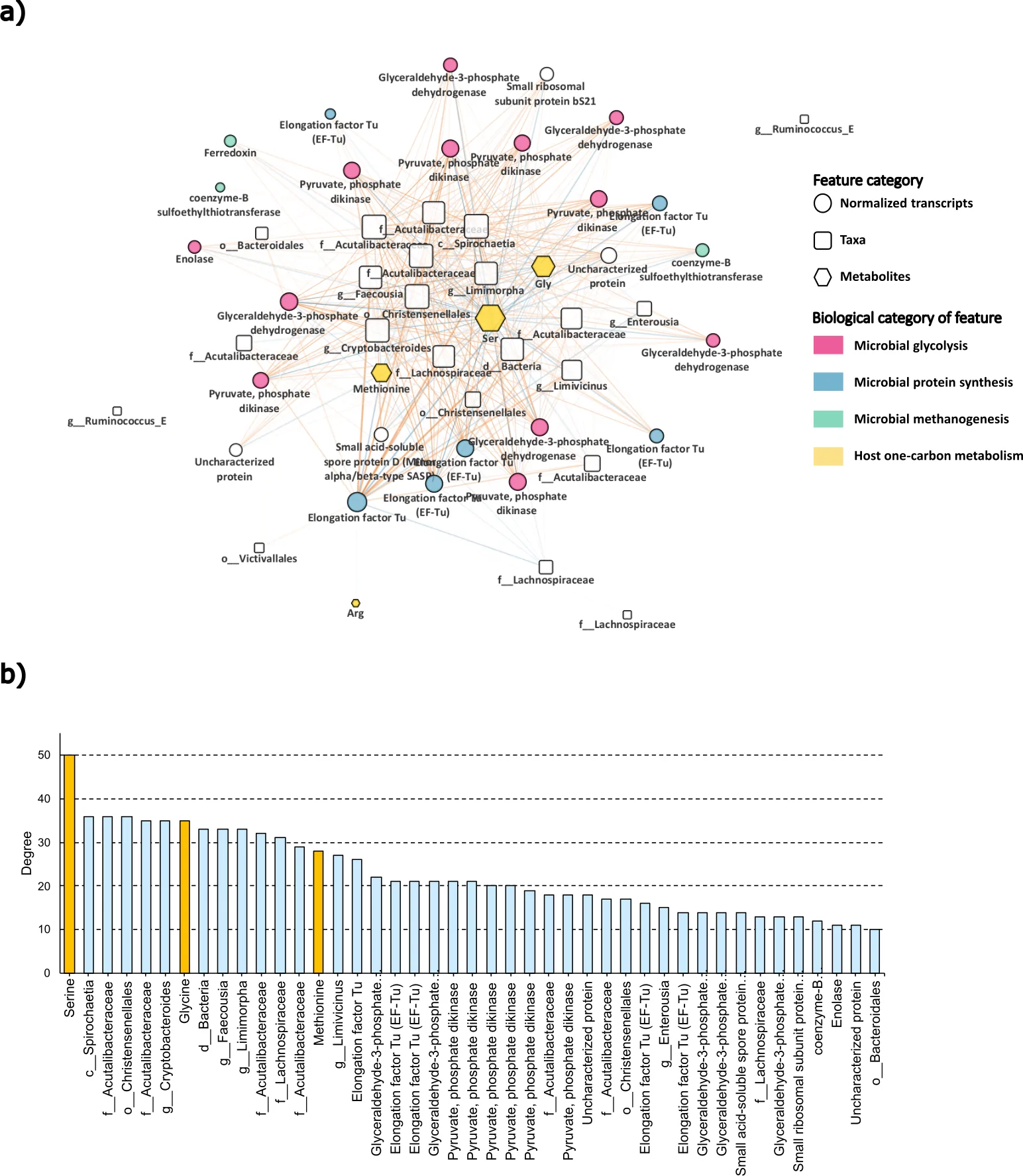
(**a**) Network of microbial normalized transcripts, taxa, and host metabolites from the multiblock sparse partial least squares analysis. Only features showing at least one absolute Pearson correlation greater than 0.6 across blocks are displayed. The size of each feature is proportional to how many other features it is connected to. Features highly correlated (|r| > 0.6) with many other features appear larger than those with fewer connections. Line colors indicate the direction of the correlation, with orange representing positive correlations and blue representing negative correlations. (**b**) Ranked distribution of feature degree (i.e., the number of edges connected to each feature) within the network. Gold bars highlight the centrality of host one-carbon metabolism features in the network.

**Fig 4:**
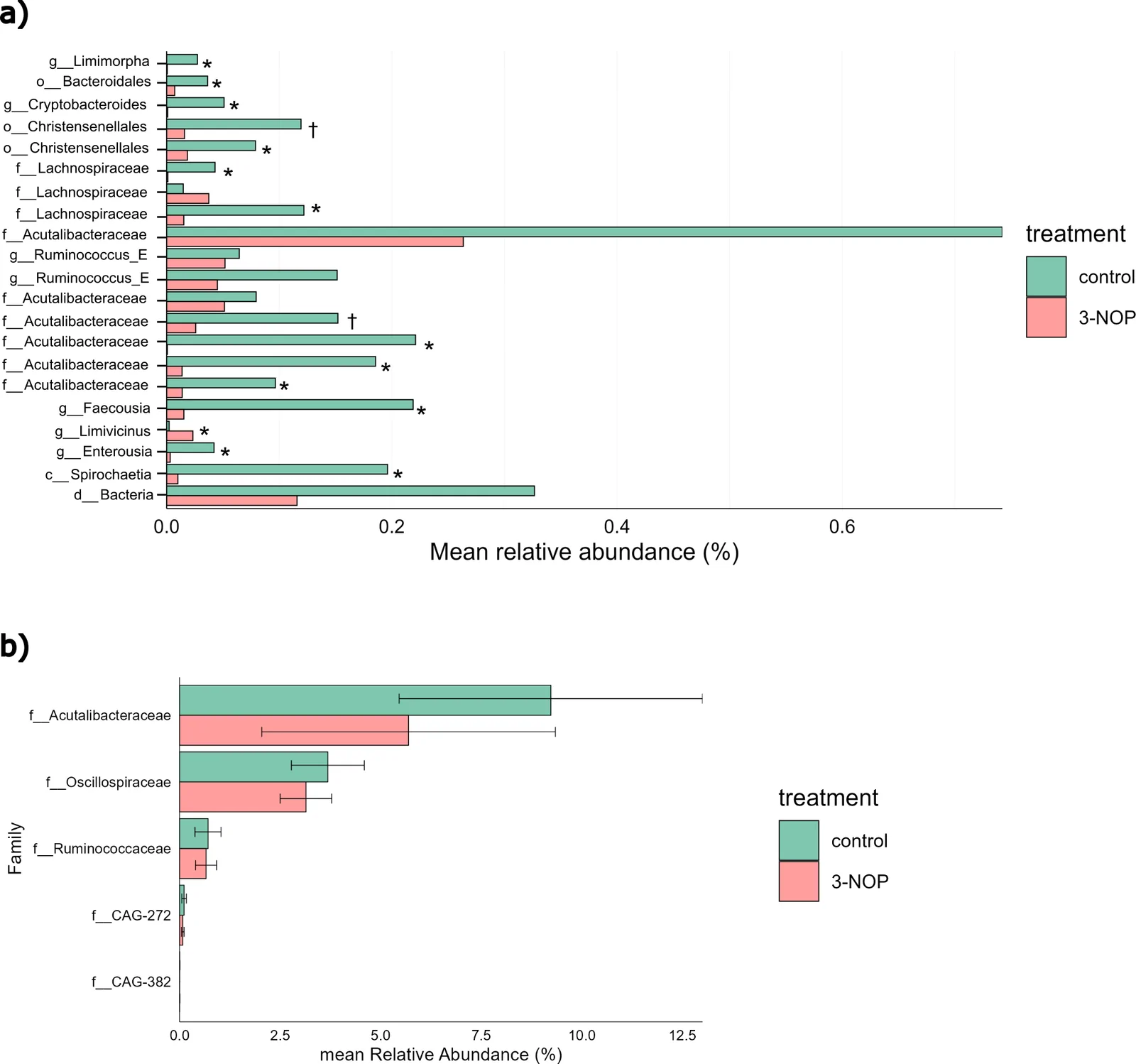
(**a**) Average relative abundance of the taxa selected in the multiblock sparse partial least squares analysis in CONTROL and 3-NOP animals. Taxa names are shown at the lowest readable taxonomic rank. * indicates a significant difference (*P*-adj < 0.05) in the relative abundance of taxa between the CONTROL and 3-NOP groups, † indicates a trend (*P*-adj < 0.1). (**b**) Average relative abundance of the o__Oscillospirales families in CONTROL and 3-NOP cows.

An MB-sPLS was performed to integrate the rumen microbiota taxonomy and activity, and the host metabolome during methanogenesis inhibition. On the two components used for the MB-sPLS, 70 and 65 taxa at the species level were retained on the first and second components, respectively, for taxonomy data, and 35 and 70 normalized transcripts were retained on the first and second components, respectively, for metatranscriptomic data. Two components were retained in the MB-sPLS model, with group differentiation occurring primarily along the first component (Fig. 2a). This component accounted for 10%, 7%, and 28% of the variance in the metataxonomic, metatranscriptomic, and metabolomic data sets, respectively. The features selected on this component, which contributed most strongly to the explanation of the metabolomic variation, are shown in Fig. 2b and c. All but one of the selected normalized transcripts were less abundant in the cows supplemented with 3-NOP compared to CONTROL cows. Selected features of the MB-sPLS were then plotted into a correlation network (Fig. 3a). The one-carbon host metabolites serine, glycine, and methionine, all in higher concentrations in the plasma of cows supplemented with 3-NOP (Fig. S3), display major centrality in the network, serine (degree = 50), glycine (d = 35), and methionine (d = 28) (Fig. 3b). The network included the normalized transcripts from microbial glycolysis (glyceraldehyde-3-phosphate dehydrogenase, pyruvate, phosphate dikinase, and enolase), methanogenesis or related features (coenzyme B sulfoethylthiotransferase a.k.a methyl coenzyme reductase and ferredoxin), and protein synthesis features (elongation factors). There were 21 correlated microbial taxa in the network that mainly belonged to the Acutalibacteraceae, Anaeroplasmataceae, and Christensenellaceae (f__CAG-138) families and to the Oscillospirales order. In all cases, except for f__Lachnospiraceae and g__Limivicinus, the taxa were in lower abundance in the 3-NOP group (Fig. 4a), with 13 of them showing significant changes due to the treatment. The relative abundance at the family level within the Oscillospirales order is shown in Fig. 4b. There were no significant differences in the relative abundance of the families, but numerical decreases were observed for Acutalibacteraceae and Oscillospiraceae families in the 3-NOP group.

### Microbial glycolysis and methanogenesis pathways tended to be downregulated in the 3-NOP group

**Fig 5:**
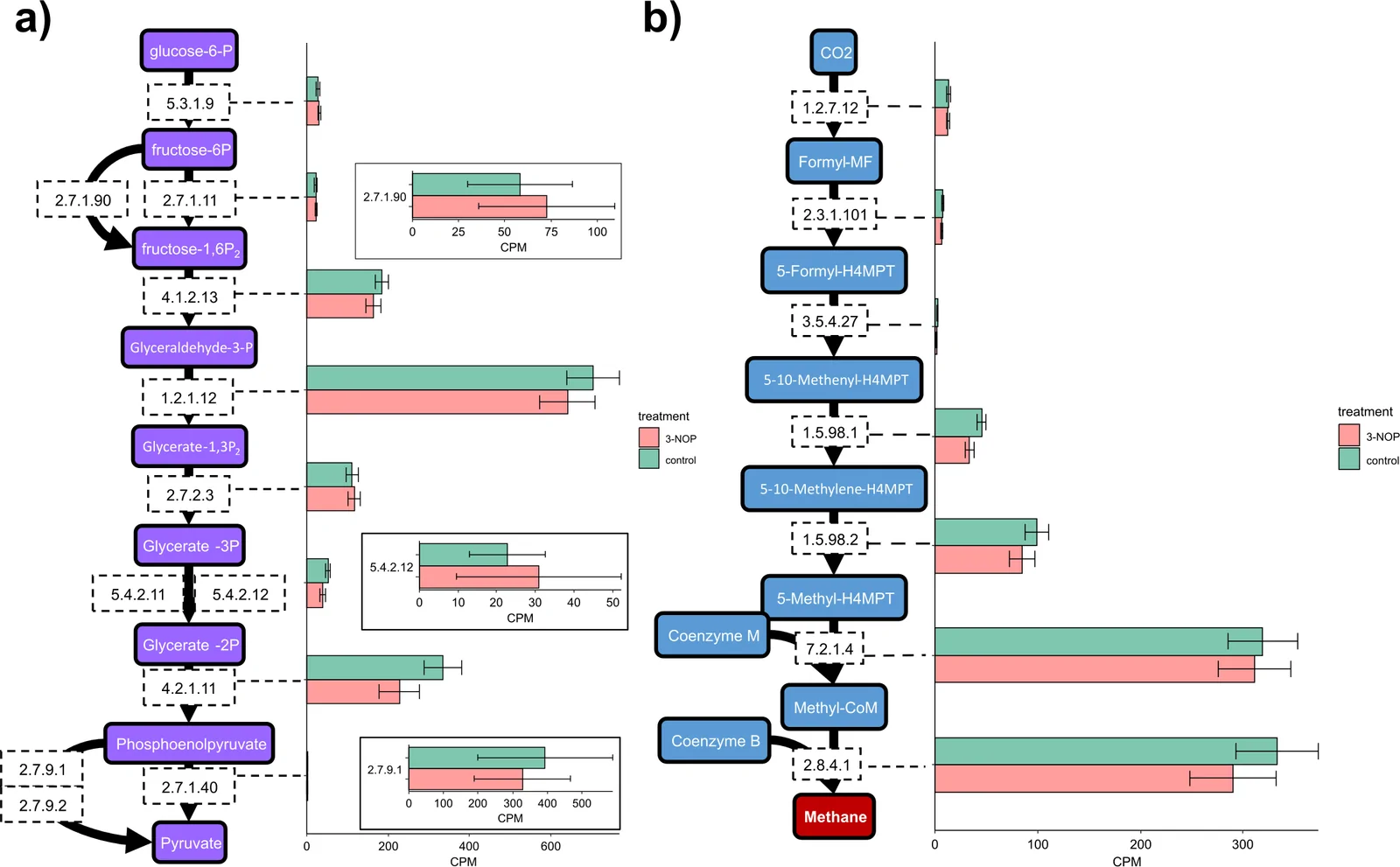
(**a**) Microbial glycolysis and (**b**) hydrogenotrophic methanogenesis pathways with the corresponding normalized abundance of the enzyme class numbers. Bar plots show the mean normalized abundance of respective transcripts with standard error.

The metabolic pathways of microbial glycolysis and hydrogenotrophic methanogenesis are presented in Fig. 5. Normalized transcript abundance for enzymes involved in hydrogenotrophic methanogenesis was numerically lower in 3-NOP-supplemented cows than in CONTROL cows, although the difference was not statistically significant. Similarly, normalized transcripts for the aforementioned enzymes in the glycolysis pathway, EC 1.2.1.12 (glyceraldehyde-3-phosphate dehydrogenase), EC 4.2.1.11 (enolase), and EC 2.7.9.1 (pyruvate and phosphate dikinase) were numerically lower in 3-NOP-supplemented cows than in CONTROL cows, although the difference was not statistically significant. The methylohydrogenotrophic methanogenesis pathway is presented in Fig. S4 but is incomplete, with two out of four mRNAs coding for the enzymes detected. The two enzymes detected, trimethylamine methyltransferase and dimethylamine methyltransferase, were numerically lower in 3-NOP cows.

## DISCUSSION

The relationship between rumen microbiota and host metabolism is central to ruminant biology. Ruminants obtain energy from feed through the microbiota in their rumen, which influences production traits and methane emissions. Specifically, inhibiting methanogenesis has proven to be an effective and safe mitigation strategy. However, further characterization of the association between the rumen microbiota and host metabolism is necessary to understand how specific methanogenesis inhibition preserves rumen fermentation, despite blocking a major H₂ sink, and how the resulting, more subtle changes affect the host. To this end, the taxonomic and functional profiles of the rumen microbiota were characterized in light of changes in the host plasma metabolome. Overall, the host plasma metabolome showed a moderate association with rumen microbiota composition and functions. A limited number of microbial taxa belonging to the order Oscillospirales, together with transcripts related to microbial glycolysis, methanogenesis, and protein synthesis, were associated with host one-carbon metabolites. Nevertheless, the mechanisms underlying this microbiota-host interaction remain to be elucidated.

The association between the rumen microbiota and the host plasma metabolome was largely conserved under methanogenesis inhibition, as shown by the MB-sPLS and multivariate analysis of individual blocks. In previous studies, specific methanogenesis inhibition partially changed the composition of the rumen microbiota depending on the time of sampling (33, 36, 37), but it did not negatively change production traits (38, 39). Several factors may explain why the association between microbiota and host plasma metabolome was moderate in this study. First, the level of methane reduction was relatively modest. Morgavi et al. (4) noted that the level of methane mitigation achieved by additives (20%–30%) may be insufficient to produce a substantial increase in metabolizable energy for the host. In the present study, the observed 22% reduction in methane yield falls at the lower end of this range. The similar concentration of total VFA in the rumen, which are the major metabolites passed to the host to supply their energy needs, is consistent with this statement, although VFA were measured before feeding. Second, the rumen microbiota activity was not at its highest across the groups at the time of sampling. The rumen microbial activity exhibits pronounced diurnal variation, with fermentation intensity and methane production typically peaking 2–3 h post-feeding (40, 41). Indeed, the abundance of microbial transcripts, responsible for fiber digestion and methane production, is higher 3 h after feeding (42). In this study, the rumen fluid was collected once in the morning, prior to the animals’ first meal. Consequently, if transcript abundance was limited due to low microbial activity, the magnitude of differences between groups was less likely to be high. It was thus difficult to determine whether the conserved association between rumen microbiota and host metabolism was due to the limited activity of rumen microbiota or the absence of a significant effect of 3-NOP. The trend of increased propionate and, to a certain extent, valerate strengthens the assumption of a moderate change in microbial activity. Indeed, it was expected that the inhibition of methanogenesis would increase propionate concentration in the rumen (5), as propionate is a major electron sink that redirects H_2_ accumulation after methanogenesis inhibition (36). Finally, the metabolomic variance was explained not only by the rumen microbiota. Indeed, the plasma metabolome reflects the full physiology of the holobiont and its interaction with the environment (43). This necessarily dilutes the fraction of the metabolomic variance explained by the rumen microbiota. The cows in this study were all in similar physiological stages; they received the same diet, and they were housed in the same conditions, which would limit, but not completely remove, influences not related to the rumen microbiota. Thus, despite previously mentioned limitations, the association between rumen microbiota and host metabolism can be explored, although the association was expected to be subtle.

Inhibition of methanogenesis by 3-NOP altered the abundance of several transcripts involved in glycolysis and methanogenesis, but not the complete metabolic pathways they are involved in. In the rumen, bacteria break down glucose and other monosaccharides before the fermentation of pyruvate. The enzyme glyceraldehyde-3-phosphate dehydrogenase (GAPDH) catalyzes the reaction between D-glyceraldehyde 3-phosphate and NAD + into 3-phospho-glyceroyl phosphate, NADH, and H^+^. These two latter compounds are used by a ferredoxin- and a NAD-dependent hydrogenase (11) to produce H_2_ and regenerate NAD^+^ for glycolysis. However, when animals are fed with 3-NOP, the main hydrogen sink, that is*,* methanogenesis, is inhibited. This increases the concentration of H_2_ in the rumen juice (33), which may be further expelled (39). In *in vitro* conditions, accumulation of H_2_ tends to inhibit the recycling of NADH into NAD^+^ because H_2_ inhibits the activity of hydrogenase coupled with ferredoxin oxidation (5, 11). In our study, we hypothesize that glycolysis was disturbed due to the accumulation of H_2_ that prevented the regeneration of NAD+. This hypothesis is supported by the lower abundance of other glycolysis transcripts in 3-NOP cows, including pyruvate phosphate dikinase and enolase transcripts, as well as the lower abundance of ferredoxin transcripts (although oxidation state is not known). However, at the glycolytic pathway level, the transcript abundance aggregated at the enzyme level was not significantly reduced in animals supplemented with 3-NOP compared to CONTROL animals. This finding, therefore, mitigates the suggestion that the NAD + cofactor was not being regenerated. The NAD^+^ to NADH ratio and dissolved H_2_ should be measured to confirm the hypothesis of disturbed glycolysis due to H_2_ accumulation. Similarly, the abundance of a number of MCR transcripts was decreased, but not the whole methanogenesis pathway. Only 17 transcripts were significantly different between treatment groups after correction for false discovery rate, indicating a limited transcriptomic signal. Therefore, pathway-level interpretations based on transcriptomic data should be considered cautiously and viewed as exploratory. This limited signal may be related to the timing of sampling relative to the microbial response to 3-NOP, although this hypothesis cannot be confirmed from the present data set. Pitta et al. also observed variation in the decrease of enzyme transcripts in the methanogenesis pathway, depending on the week of sampling (33), which strengthens the dependence of the 3-NOP effect on the microbial pathway over time. Otherwise, methanogenesis inhibition may have impacted only specific bacterial populations, which could explain the selective reduction in, at least, the glycolytic transcripts instead of a community-wide decline.

Cows supplemented with 3-NOP showed a specific reduction of the relative abundance of the families Acutalibacteraceae, Oscillospiraceae, CAG-138 (from the Christensenellaceae order), and one Ruminococcaceae taxon. These taxa are all hydrogen producers and have been associated with methanogens in previous publications. For instance, the Acutalibacteraceae Candidatus Vesiculincola pelomyxae live in syntrophy with the methanogen Candidatus Methanoregula pelomyxae, within the eukaryote Pelomyxa schiedti (44). Oscillospiraceae have been associated with methanogens in fecal microbiota centenarian (45), while Christensenellaceae, also associated with methanogens in the same study, exhibit physical association with *Methanobrevibacter smithii* (46). The reduction in the abundance of elongation factor transcripts, all but one attributed to the Eubacteriales order, is consistent with the decrease in abundance of taxa belonging to the Acutalibacteraceae and Oscillospiraceae families. Elongation factors are prokaryotic proteins involved in protein synthesis by binding and carrying acyl-tRNA to the ribosome during translation (47). The proteins themselves represent 5%–10% of the total cell protein (48) and are widely represented in the rumen proteome, up to 44% in a metaproteomic study (49). Reduction in elongation factors then suggests a downregulation in the translation machinery, potentially a reduced protein synthesis capacity or a shift in cell priorities. Thus, it is possible that reduced methanogenic activity, as shown by the reduction of several MCR transcripts, has reflected on these syntrophic bacteria. Interestingly, there were no differences in Archaea abundance between the control and 3-NOP groups. This suggests that the response to 3-NOP was driven more by changes in methanogen activity than by changes in archaeal population size, in agreement with previous studies showing that 3-NOP reduces methanogenic activity rather than methanogen abundance (33, 35). As stated in previous paragraphs, 3-NOP favors H_2_ concentration in the rumen. It is possible that an increase in H_2_ concentration has led to a decrease in hydrogen producers (11, 50), although this phenomenon is not verified for all rumen species. In addition, the rumen fluid was sampled once during the trial, which did not allow us to evaluate how the microbiota composition, especially the hydrogen producers, changed over time due to 3-NOP supplementation. A dynamic assessment of the rumen microbiota along the host metabolome would have been valuable to decipher whether microbiota composition variation is mirrored in the plasma metabolome.

In the previous publication of our group, discriminant metabolites between animals supplemented with or without 3-NOP were identified. In the present study, these metabolites were used to explore their association with taxa and transcripts from the rumen microbiota, thereby providing insight into the effect of methanogenesis inhibition on the ruminant holobiont. The association between rumen microbiota and plasma metabolome following the inhibition of methanogenesis was related to host one-carbon metabolites. One-carbon metabolism is a metabolic pathway that synthesizes nucleic acids and furnishes methyl groups for methylation reactions such as the synthesis of choline derivatives. In dairy cattle, the need for methyl groups is high during the transition from gestation to lactation to incorporate choline and proteins into milk (51). Two hypotheses might be proposed to explain the specific link of rumen microbiota to host one-carbon metabolites in our case. The first hypothesis is that the methyl intermediate compounds of the methanogenesis pathway accumulate when 3-NOP inhibits the MCR enzyme. A majority of rumen methanogens exhibit a serine hydroxymethyltransferase in their genome (Fig. S5) capable of transforming methanogenesis metabolite intermediates into serine. However, the transcript abundance of serine hydroxymethyltransferase, despite being numerically higher in the 3-NOP group (data not shown), was not significantly modified by the 3-NOP. The second hypothesis is that disturbances in microbial glycolysis cause an accumulation of the intermediate 3-phosphoglycerate, which serves as a precursor for serine (51). However, as already shown, the whole glycolytic pathway was not significantly reduced in animals supplemented with 3-NOP. The main limitation in developing these hypotheses in this study is that only VFA were analyzed as rumen metabolites. Consequently, these two hypotheses should be further assessed by the quantification of metabolite intermediates in these two pathways in rumen fluid. Finally, an untargeted metabolomic assessment of the rumen fluid would have helped us to establish more precise mechanisms of interaction between rumen microbiota and host metabolism, given that microbial metabolites act as intermediate mediators within the holobiont (52).

### Conclusion

In this study, we explored the association between the rumen microbiota and the host metabolome when methanogenesis was inhibited. Overall, the inhibition of methanogenesis did not appear to substantially alter the association between rumen microbiota and plasma metabolites. However, reduced relative abundance of several transcripts involved in microbial glycolysis and protein synthesis, and the increase in one-carbon metabolites in the host metabolome led to the formulation of multiple hypotheses that remain to be tested. In particular, based on the observations of this study, we hypothesize that inhibiting methanogenesis disrupts microbial glycolysis and protein synthesis, particularly in specific hydrogen producers. We could not determine a precise mechanism linking rumen microbiota and one-carbon metabolites. However, given the importance of methyl donors in fueling one-carbon metabolism in the host, we suggest investigating the accumulation of methyl compounds in the rumen and their subsequent transfer to the host.

## Data Availability

Metagenomic and metatranscriptomic data are available under project PRJEB96328 in the European Nucleotide Archive. Discriminant metabolite relative quantifications are available in Data S1.

## SUPPLEMENTAL MATERIAL

Data S1 spectrum.00269-26-s0001.txt Metabolomic data.

Supplemental tables and figures spectrum.00269-26-s0002.docx Tables S1 to S4 and Fig. S1 to S5.

ASM does not own the copyrights to Supplemental Material that may be linked to, or accessed through, an article. The authors have granted ASM a non-exclusive, world-wide license to publish the Supplemental Material files. Please contact the corresponding author directly for reuse.
