# Supplementary material for: Integrative analysis of rumen microbiota activity and host metabolism following methanogenesis inhibition in dairy cattle
Source: Microbiol Spectr. 2026 Jul 23;14(9):e00269-26. doi: 10.1128/spectrum.00269-26 (PMC13532202; doi:10.1128/spectrum.00269-26)
Supplement: Supplementary file 1 [file spectrum.00269-26-s0002.docx]

**Supplementary Table 1:** Metagenomics paired-end reads number before and after pre-processing.

| Sequence | Sample_id | Treatment | Initial number of paired-end reads | Number of paired-end reads after quality filtering | Number of paired-end reads after decontamination | Percentage of paired-end reads retained |
| --- | --- | --- | --- | --- | --- | --- |
| mgx | 150N | 3NOP | 109025685 | 100585325 | 90152423 | 82.7 |
| mgx | 151N | 3NOP | 34640669 | 31596181 | 29248730 | 84.4 |
| mgx | 152T | CONTROL | 84410770 | 78278058 | 71317864 | 84.5 |
| mgx | 153T | CONTROL | 30780241 | 27832962 | 25318898 | 82.3 |
| mgx | 155T | CONTROL | 86256697 | 80007115 | 73738557 | 85.5 |
| mgx | 156N | 3NOP | 32384042 | 29170581 | 26560263 | 82.0 |
| mgx | 159N | 3NOP | 93454908 | 86230596 | 78362582 | 83.9 |
| mgx | 165N | 3NOP | 33520602 | 30330985 | 27712077 | 82.7 |
| mgx | 166T | CONTROL | 34727424 | 31009410 | 27731225 | 79.9 |
| mgx | 167T | CONTROL | 92895458 | 85518006 | 78750610 | 84.8 |
| mgx | 168T | CONTROL | 87635036 | 80236220 | 68725018 | 78.4 |
| mgx | 170N | 3NOP | 31126344 | 28241690 | 25186718 | 80.9 |
| mgx | 172T | CONTROL | 31239322 | 28149132 | 25818113 | 82.6 |
| mgx | 178N | 3NOP | 96979070 | 88998469 | 78878365 | 81.3 |
| mgx | 180N | 3NOP | 36068805 | 32523745 | 29878232 | 82.8 |
| mgx | 600N | 3NOP | 30135733 | 27487398 | 25672887 | 85.2 |
| mgx | 601T | CONTROL | 89334764 | 82460580 | 75697043 | 84.7 |
| mgx | 890N | 3NOP | 30567230 | 27535754 | 25672578 | 84.0 |
| mgx | 892T | CONTROL | 33517878 | 30136112 | 27344097 | 81.6 |
| mgx | 900T | CONTROL | 33248346 | 30188813 | 27419126 | 82.5 |
| mgx | 913N | 3NOP | 31040651 | 28182736 | 26265377 | 84.6 |
| mgx | 918T | CONTROL | 31073878 | 27808557 | 24445734 | 78.7 |
| mgx | 937T | CONTROL | 30252346 | 27245769 | 25151855 | 83.1 |
| mgx | 938N | 3NOP | 37844584 | 33875791 | 30738833 | 81.2 |
| mgx | 941T | CONTROL | 31191072 | 28489551 | 25892857 | 83.0 |
|  |  | average | 51734062.2 | 47284781.4 | 42867202.5 | 82.7 |
|  |  | sd | 28328693.2 | 26391512.2 | 23676287.2 | 1.9 |

**Supplementary Table 2:** Metatranscriptomics paired-end reads number before and after pre-processing.

| Sequence | Sample_id | Treatment | Initial number of reads | Number of reads after quality filtering | Number of reads after rRNA removal | Percentage of reads retained |
| --- | --- | --- | --- | --- | --- | --- |
| mtx | 150N | 3NOP | 83195479 | 73634029 | 3592428 | 4.3 |
| mtx | 151N | 3NOP | 84756391 | 74931006 | 4098776 | 4.8 |
| mtx | 152T | CONTROL | 76133034 | 66903290 | 3235947 | 4.3 |
| mtx | 153T | CONTROL | 88663493 | 78978960 | 3738265 | 4.2 |
| mtx | 155T | CONTROL | 88918316 | 77597143 | 4658564 | 5.2 |
| mtx | 156N | 3NOP | 97860104 | 86619939 | 3912748 | 4.0 |
| mtx | 159N | 3NOP | 93321848 | 82406545 | 4025321 | 4.3 |
| mtx | 165N | 3NOP | 83817446 | 73303981 | 3544051 | 4.2 |
| mtx | 166T | CONTROL | 78131190 | 69841584 | 3793154 | 4.9 |
| mtx | 167T | CONTROL | 93619086 | 82451217 | 3738918 | 4.0 |
| mtx | 168T | CONTROL | 92477862 | 82084910 | 3597111 | 3.9 |
| mtx | 170N | 3NOP | 97590311 | 86092140 | 3411080 | 3.5 |
| mtx | 172T | CONTROL | 87171548 | 77735794 | 3605687 | 4.1 |
| mtx | 178N | 3NOP | 75571982 | 67343342 | 3191325 | 4.2 |
| mtx | 180N | 3NOP | 81512595 | 70069197 | 3465063 | 4.3 |
| mtx | 600N | 3NOP | 74017143 | 63706051 | 2622746 | 3.5 |
| mtx | 601T | CONTROL | 80822023 | 71554816 | 4479905 | 5.5 |
| mtx | 890N | 3NOP | 75900233 | 67774634 | 4236419 | 5.6 |
| mtx | 892T | CONTROL | 76052963 | 67760378 | 3314715 | 4.4 |
| mtx | 900T | CONTROL | 107224942 | 94410622 | 4287004 | 4.0 |
| mtx | 913N | 3NOP | 94790613 | 83742506 | 4924593 | 5.2 |
| mtx | 918T | CONTROL | 80878740 | 71610100 | 3507388 | 4.3 |
| mtx | 937T | CONTROL | 95039392 | 84188916 | 4297614 | 4.5 |
| mtx | 938N | 3NOP | 77852302 | 69229459 | 3833494 | 4.9 |
| mtx | 941T | CONTROL | 88879601 | 79150344 | 3869153 | 4.4 |
|  |  | average | 86167945.5 | 76124836.1 | 3799258.8 | 4.4 |
|  |  | sd | 8553283.2 | 7591071.5 | 496074.4 | 0.5 |

**Supplementary Table 3:** Methane and milk production in lactating dairy cows supplemented with 3-NOP (3-NOP, n = 12) or without (CONTROL, n= 13).

|  | CONTROL | | | 3-NOP | | |  |
| --- | --- | --- | --- | --- | --- | --- | --- |
|  | average | | sd | average | | sd | P-value |
| Methane (g/d) | 334.9 | | 41.8 | 259.0 | | 51.1 | 6.10^-3^ |
| Methane intensity (g/kg milk) | 29.7 | | 3.2 | 23.4 | | 3.9 | 3.10^-3^ |
| Methane yield (g/kg DMI) | 17.4 | | 2.0 | 13.6 | | 1.7 | 9.10^-4^ |
| Milk yield (kg/d) | 23.6 | | 3.0 | 24.6 | | 3.26 | 0.45 |
| Milk yield DMI ratio | 1.3 | | 0.2 | 1.3 | | 0.1 | 0.46 |

**Supplementary Table 4:** Normalized transcripts that were significantly different (FDR < 0.05 ) or tend to be different (FDR < 0.1) between control and 3-NOP groups.

| feature | protein_name | value | coef | stderr | qval |
| --- | --- | --- | --- | --- | --- |
| UniRef90_K0T444 | Histone H4 | control | 1.34 | 0.17 | 0.003 |
| UniRef90_A0A126QW78 | Methyl-coenzyme M reductase subunit beta | control | 2.60 | 0.36 | 0.004 |
| UniRef90_A0A0F0CD99 | Glyceraldehyde-3-phosphate dehydrogenase | control | 0.56 | 0.09 | 0.013 |
| UniRef90_A0A395ZZQ9 | deleted | control | 0.82 | 0.14 | 0.037 |
| UniRef90_A0A356AKT1 | Elongation factor Tu | control | 0.53 | 0.09 | 0.037 |
| UniRef90_A0A1C7GNK7 | Elongation factor Tu (EF-Tu) | control | 1.31 | 0.23 | 0.037 |
| UniRef90_A0A2V2GRF9 | deleted | control | 2.13 | 0.38 | 0.039 |
| UniRef90_UPI000C06960D | #N/A | control | 1.24 | 0.22 | 0.039 |
| UniRef90_A0A126QV28 | coenzyme-B sulfoethylthiotransferase | control | 2.18 | 0.40 | 0.039 |
| UniRef90_A0A126QVA5 | Methyl-coenzyme M reductase subunit gamma | control | 2.51 | 0.46 | 0.039 |
| UniRef90_R6CAD6 | Elongation factor Tu (EF-Tu) | control | 0.94 | 0.17 | 0.040 |
| UniRef90_A0A3D2HTT5 | Elongation factor Tu | control | 0.91 | 0.17 | 0.046 |
| UniRef90_A8SSF3 | deleted | control | 1.62 | 0.31 | 0.046 |
| UniRef90_R7G3Z3 | deleted | control | 0.41 | 0.08 | 0.046 |
| UniRef90_A0A3B8UAM2 | CDP-diacylglycerol--glycerol-3-phosphate 3-phosphatidyltransferase | control | -1.99 | 0.39 | 0.046 |
| UniRef90_A0A182VCT1 | Histone H4 | control | 1.40 | 0.27 | 0.050 |
| UniRef90_M9S9U8 | Methyl-coenzyme M reductase subunit beta | control | 2.21 | 0.44 | 0.050 |
| UniRef90_F2NSX6 | Glyceraldehyde-3-phosphate dehydrogenase | control | 0.36 | 0.07 | 0.065 |
| UniRef90_A0A0Q4BCJ2 | coenzyme-B sulfoethylthiotransferase | control | 1.92 | 0.40 | 0.073 |
| UniRef90_W6TZP8 | Histone H4 | control | 1.71 | 0.36 | 0.087 |
| UniRef90_A0A349DDB8 | Ferredoxin | control | 1.80 | 0.38 | 0.087 |
| UniRef90_A0A1G4QFG6 | Small ribosomal subunit protein bS21 | control | 1.75 | 0.37 | 0.087 |
| UniRef90_UPI0004851457 | #N/A | control | -2.27 | 0.49 | 0.087 |
| UniRef90_A0A3D0M454 | Elongation factor Tu | control | 0.70 | 0.15 | 0.087 |
| UniRef90_A0A1I3SFN5 | deleted | control | 1.14 | 0.24 | 0.087 |
| UniRef90_A0A354U7Q5 | Ribosomal protein | control | 2.09 | 0.45 | 0.087 |

**Supplementary Figure 1:** Principal component analysis of rumen microbiota subset on Archaea in the 25 dairy cows supplemented or not with 3-NOP.

**Supplementary Figure 2:** Principal component analysis of normalized transcripts of rumen microbiota in the 25 dairy cows supplemented or not with 3-NOP.

**Supplementary Figure 3:** Whisker-plot of one-carbon metabolites serine, glycine and methionine in the host plasma metabolome of the 25 dairy cows supplemented or not with 3-NOP.

**Supplementary Figure 4:** Normalized transcripts abundance of methylohydrogenotrophic methanogenesis enzyme classes in the 25 dairy cows supplemented or not with 3-NOP. Dashed lines with no bar plot indicate the enzymes EC number was not found in our dataset.

**Supplementary Figure 5:** Phylogenetic tree of A) Archaea and B) selected genus of Methanobacteria colored by Family. Red lines depict the presence of a serine hydroxymethyltransferase in the genome of Archaea. Genereated with Annotree (Mendler, K., Chen, H., Parks, D.H., Lobb, B., Hug, L.A., Doxey, A.C., 2019. AnnoTree: visualization and exploration of a functionally annotated microbial tree of life. Nucleic Acids Research 47, 4442–4448. <https://doi.org/10.1093/nar/gkz246>)
